# The Use of Radiotherapy in Leptomeningeal Carcinomatosis: A Systematic Review and Random-Effects Proportions Meta-Analysis

**DOI:** 10.3390/cancers18040547

**Published:** 2026-02-07

**Authors:** Pamela Ochoa-Lantigua, Mauricio Moreno-Bejarano, Cayetana Guarderas-Arias, José Bueno-Miño, Jose E. Leon-Rojas

**Affiliations:** 1 NeurALL Research Group, Quito EC170157, Ecuador; maochoala@uide.edu.ec (P.O.-L.); mauricio.moreno.bejarano@udla.edu.ec (M.M.-B.); 2 Escuela de Medicina, Universidad de Las Américas, Quito EC170124, Ecuador; cayetana.guarderas@udla.edu.ec (C.G.-A.); jose.bueno@udla.edu.ec (J.B.-M.); 3 Grupo de Investigación Bienestar, Salud y Sociedad, Escuela de Psicología y Educación, Universidad de Las Américas, Quito EC170124, Ecuador

**Keywords:** leptomeningeal carcinomatosis, radiotherapy, toxicity, survival, whole-brain radiotherapy, craniospinal irradiation

## Abstract

**Objective**: Leptomeningeal carcinomatosis (LMC) is a rare but devastating complication of advanced cancer, particularly in patients with breast and lung malignancies. This systematic review provides a descriptive synthesis of radiotherapy approaches used in patients with leptomeningeal metastases, with a quantitative proportions meta-analysis focused on treatment-related toxicity. **Materials and methods**: A systematic search was conducted in PubMed, Scopus, and the virtual health library (BVS) databases following PRISMA 2020 guidelines. Studies including patients diagnosed with LMC and treated with RT were selected. Outcomes included overall survival (OS), adverse events (toxicities), and functional response. **Results**: A total of 39 studies comprising 2822 patients were included; the most frequent primary tumors were lung (*n* = 1337) and breast (*n* = 990) cancers. The mean time from cancer diagnosis to LMC was 22.4 months. Radiotherapy regimens included whole-brain radiotherapy (WBRT, *n* = 1054), craniospinal irradiation (CSI, *n* = 148), and focal RT (*n* = 27); RT was administered alone or in combination with systemic treatments. Toxicity was reported in 462 patients, primarily fatigue (*n* = 115), nausea/vomiting (*n* = 72), and hematological events (notably in CSI). The pooled toxicity prevalence was 50.8% (95% CI, 26.1–75.4; I^2^ = 96.1; *p* < 0.0001) for all RT modalities, and 31.6% (95%CI, 15.0–50.8; I^2^ = 90.7; *p* < 0.0001) for WBRT. CSI toxicity estimates were based on a limited number of studies and did not reach statistical significance, and should therefore be interpreted as exploratory. Mean OS from LMC diagnosis was 18.2 weeks; OS by treatment was 21.5 weeks and 20.3 weeks, for RT by itself and combined, respectively. **Conclusions**: LMC predominantly affects patients with advanced-stage lung and breast cancers and presents with variable clinical timelines and functional impairment. Radiotherapy represents a frequently utilized and clinically important component of the palliative management of leptomeningeal disease, particularly for symptom control and neurological stabilization, rather than a treatment associated with superior survival outcomes. Prognosis is more closely linked to patient-specific factors than to treatment type. Radiotherapy toxicity is prevalent; however, most are categorized as type 1 toxicities with insignificant to little damaging effects on patients.

## Simple Summary

Leptomeningeal carcinomatosis is a serious complication of advanced cancer in which cancer cells spread to the membranes surrounding the brain and spinal cord, often causing rapid neurological decline and very limited survival. Radiotherapy is frequently used to relieve symptoms, but its real benefits and side effects have not been clearly defined. In this study, we systematically reviewed the published research to better understand how radiotherapy is used in this condition, how long patients survive after treatment, and what side effects may occur. By analyzing data from more than 2800 patients, we found that radiotherapy remains important for symptom control, although it does not clearly extend survival, and that different radiotherapy approaches carry different risks of side effects. These findings help clarify the role of radiotherapy in leptomeningeal carcinomatosis and may guide clinicians and researchers toward more informed, patient-centered treatment decisions.

## 1. Introduction

Leptomeningeal carcinomatosis (LMC) is a condition that originates from the spread of malignant cancer cells to the leptomeninges, originating mostly from breast cancer (12–25%), lung cancer (10–26%), and melanoma (5–24%) [1]. It is a current public health concern due to its increased incidence over the past few years (probably due to rise in the precision of diagnoses), as well as its limited overall survival (OS) rate and poor prognosis [2]. Median OS after diagnosis currently ranges between 10 and 15 weeks [3]; more specifically, 14 weeks for lung cancer, 7 weeks for gastrointestinal cancers, and 12 weeks for breast cancer [4]. LMC management is challenging due to the lack of effectiveness of treatment options such as chemotherapy due to its inefficient penetration of the blood–brain barrier [5]. Moreover, intrathecal chemotherapy has shown to have neurotoxicity rates of up to 47%, limiting its success as a therapeutic option [5].

In the treatment of leptomeningeal carcinomatosis, clinical guidelines such as the EANO–ESMO Clinical Practice Guideline highlight the importance of analyzing key factors like patient functional status (Karnofsky ≥ 60), neurological symptoms, and the dissemination of the disease outside the brain [6]. In these guidelines, radiotherapy has become an important tool to manage neurological symptoms and treat LMC; whole-brain radiotherapy (WBRT) and focal spinal radiotherapy are commonly used as palliative treatments but have not shown any improvement in patients’ OS [6,7,8]. Despite widespread use, the impact of radiotherapy on survival outcomes in LMC remains uncertain, and existing evidence is derived largely from retrospective series with heterogeneous patient populations and treatment protocols [7,8]. Moreover, radiotherapy-associated toxicity, especially hematological toxicity related to CSI, represents a significant clinical concern and may limit treatment eligibility in patients with poor baseline performance status or extensive systemic disease [7]. Consequently, a clear understanding of the balance between potential symptomatic benefit, survival outcomes, and treatment-related adverse effects is essential to inform clinical decision-making in this vulnerable population.

Although radiotherapy is widely regarded as a palliative intervention in leptomeningeal metastases, its clinical use extends across a broad range of scenarios, including symptom control, cerebrospinal fluid flow restoration, and neurological stabilization [6,7,8]. Despite its frequent application, the evidence base guiding radiotherapy decision-making remains fragmented. Beyond the general acknowledgement of its palliative intent, what remains insufficiently defined is the toxicity burden associated with different radiotherapy approaches, the variability of adverse events across treatment strategies, and the extent to which these toxicities may compromise neurological function and quality of life in a population with limited life expectancy. Existing studies report highly heterogeneous efficacy-related outcomes, including overall survival, neurological response, and symptom control, using inconsistent definitions and time points. This heterogeneity precludes robust comparative effectiveness analyses and limits the interpretability of pooled survival estimates. In contrast, treatment-related toxicity is more consistently reported across studies, yet has not been systematically quantified despite its central relevance to clinical decision-making in this fragile population.

For these reasons, our study was designed to provide a comprehensive descriptive synthesis of radiotherapy outcomes in leptomeningeal metastases, complemented by a proportions meta-analysis focused specifically on toxicity. This approach allows the estimation of the overall burden of adverse effects associated with radiotherapy, while avoiding inappropriate comparative or time-to-event analyses unsupported by the structure of the available data. By clarifying the toxicity landscape, this work aims to inform patient counseling, risk–benefit assessment, and the design of future prospective studies.

## 2. Materials and Methods

This systematic review was performed following the Cochrane Collaboration and the Preferred Reporting Items for Systematic Reviews and Meta-Analysis (PRISMA) 2020 recommendations [9]. This review was not registered in PROSPERO, as at the time of protocol development PROSPERO did not accept registrations for reviews primarily focused on prevalence or proportion-based meta-analyses of toxicity outcomes; nevertheless, the study followed a prespecified protocol and was conducted in accordance with PRISMA guidelines.

### 2.1. Information Sources and Search Strategy

We queried PubMed, Scopus, and the Latin American and Caribbean “Biblioteca Virtual en Salud” (BVS) from inception to 17 October 2022. The main key terms used included “meningeal carcinomatosis”, “radiotherapy”, “tomotherapy”, “helicoidal therapy”, “outcome”, “toxicity”, and “survival”; a combination of free-text and medical subheading (MeSH) terms were used when feasible.

### 2.2. Eligibility Criteria

Inclusion criteria considered case series, retrospective and prospective cohort studies, clinical trials, and case–control studies that analyzed the treatment of patients with a diagnosis of leptomeningeal carcinomatosis, with at least a portion of the participants receiving radiotherapy as part of their treatment scheme. Leptomeningeal disease was defined according to the diagnostic criteria used in the original studies. Eligible studies included patients diagnosed with LMD based on cerebrospinal fluid cytology, characteristic contrast-enhanced MRI findings, or a combination of both. Given the known limitations of cytological sensitivity and the widespread clinical reliance on MRI for diagnosis, no requirement for mandatory cytological confirmation was imposed. This approach was chosen to reflect real-world diagnostic practice and to avoid excluding contemporary studies that rely primarily on radiographic criteria. The articles also had to include at least one of the outcomes of interest that included overall survival (OS), effectiveness, and adverse effects (toxicities). No limits on date, age, sex, or ethnicity were implemented and articles written in English or Spanish were accepted. The exclusion criteria included patients without a clear diagnosis of leptomeningeal carcinomatosis, or those treated without radiotherapy; those where radiotherapy was used to treat the primary tumor instead of the LMC were also excluded. Case reports, reviews, letters to the editor and commentaries were excluded.

### 2.3. Selection Process and Data Items

Two blinded authors independently performed a first screening of titles, abstracts, and keywords against the aforementioned eligibility criteria; a third author reviewed and resolved the discrepancies through discussion and mutual consensus. The second screening involved a full-text review by two independent and blinded reviewers; discrepancies were solved in a similar fashion as with the first screening. Both screenings were performed using the online software Rayyan (https://www.rayyan.ai/), specifically created for systematic reviews to minimize human error and conduct strict filtering methodologies.

Data from the selected articles were extracted by four authors in an Excel spreadsheet table for further analysis. The variables extracted included article metadata (authors, year, DOI, study design, country), demographic information (number of participants, age), primary cancer type (i.e., location of the primary tumor), survival (OS), time from cancer diagnosis to LMC, type of treatment (chemotherapy and radiotherapy or radiotherapy alone), type of radiotherapy (WBRT, CSI, other), and type of radiotherapy toxicity. For the proportion meta-analysis we extracted, when available and feasible, the absolute frequencies of the type of toxicities reported in WBRT and CSI radiotherapies; when studies did not provide the absolute frequencies or when the frequencies could not be correctly extracted to reflect patient counts rather than toxicity counts (i.e., a patient could have more than one toxicity), they were not considered for our proportions meta-analysis.

### 2.4. Risk of Bias

Risk of bias assessment was conducted using the National Heart, Lung and Blood Institute (NHLBI) study quality assessment instruments (https://www.nhlbi.nih.gov/health-topics/study-quality-assessment-tools, accessed on 15 November 2025). Each included study was independently evaluated by two reviewers, with disagreements solved by a third reviewer to reach consensus. Risk of bias was determined through the application of a 14-item, design-specific questionnaire independently completed by two reviewers. Studies were subsequently categorized as having low, moderate, or high risk of bias according to the proportion of affirmative responses, defined as ≥80% for low risk, 50–79% for moderate risk, and <50% for high risk.

### 2.5. Data Synthesis and Analysis

Due to substantial heterogeneity and inconsistent reporting of efficacy outcomes, quantitative meta-analysis was restricted to toxicity endpoints, while efficacy-related outcomes were synthesized descriptively; survival outcomes were synthesized descriptively without quantitative pooling, as key baseline prognostic variables, including patient performance status, neurological burden, and systemic disease control, were inconsistently reported across studies, precluding meaningful adjustment or comparability. Data related to survival times, time to LMC, primary tumor types, demographic characteristics, response to treatment, and specific toxicities were summarized using absolute and relative frequencies based on the total of individuals included in our systematic review. To further analyze the adverse effects of radiotherapy in LMC, we dichotomized the presence of toxicity in order to calculate the absolute frequencies of such instances and used them for a proportions meta-analysis. We used a random-effects framework, with variance stabilization via the Freeman–Tukey double arcsine transformation; summary estimates were expressed as pooled proportions with corresponding 95% confidence intervals. Statistical heterogeneity was assessed using the I^2^ statistic and interpreted as low when I^2^ was below 25%, moderate when ranging from 25% to 74%, and high when equal to or exceeding 75%. When marked heterogeneity was identified, sensitivity analyses were undertaken to investigate potential contributors to interstudy variability. Publication bias was evaluated qualitatively and incorporated into the overall risk of bias judgment, given that standard quantitative methods such as funnel plots and Egger regression are unsuitable for proportions meta-analyses and may generate misleading inferences in this setting. Meta-regression and further subgroup analyses were not performed due to the limited number of contributing studies, inconsistent reporting of key covariates, and the high risk of unstable or spurious estimates. All analyses were conducted using the Joanna Briggs Institute System for the Unified Management, Assessment and Review of Information (JBI SUMARI), Adelaide, Australia.

## 3. Results

### 3.1. Study Selection

**Figure 1:**
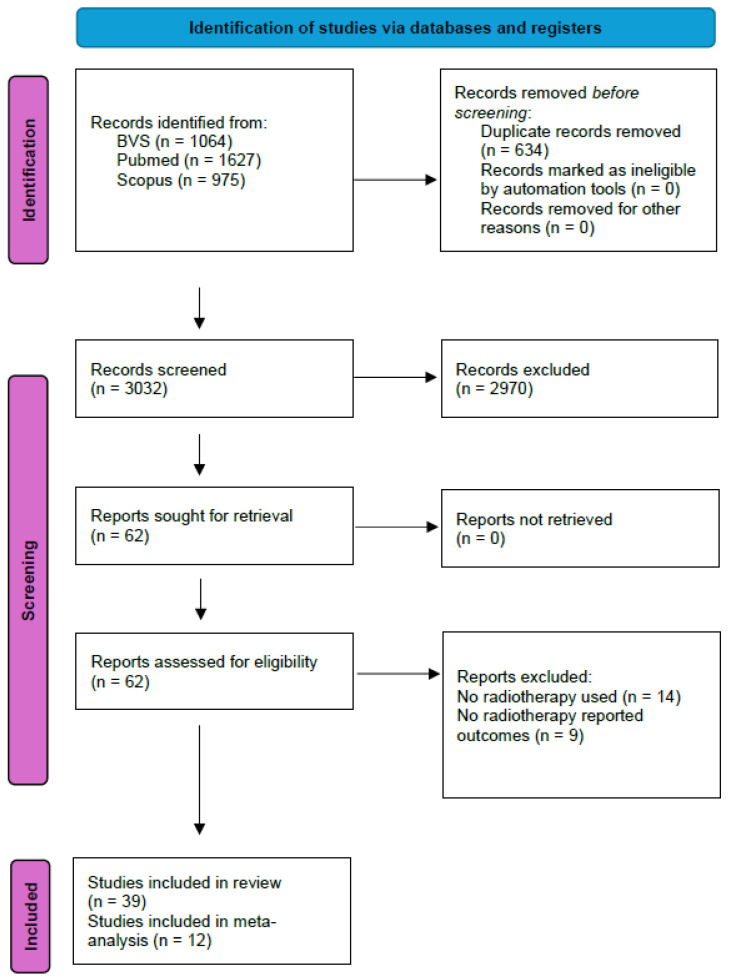
PRISMA 2020 flow diagram showcasing the selection process.

Our search yielded a total of 3302 articles, of which 62 were chosen for full-text review. Finally, after bias assessment, 39 articles were selected with a total of 2822 patients [1,2,3,4,5,10,11,12,13,14,15,16,17,18,19,20,21,22,23,24,25,26,27,28,29,30,31,32,33,34,35,36,37,38,39,40,41,42,43]. The main reasons for exclusion were the lack of radiotherapy as a part of the therapeutic scheme or for not clearly reporting an outcome of radiotherapy use. The complete filtering process can be seen in Figure 1.

### 3.2. Risk of Bias

Risk of bias analysis of the 39 included articles [1,2,3,4,5,10,11,12,13,14,15,16,17,18,19,20,21,22,23,24,25,26,27,28,29,30,31,32,33,34,35,36,37,38,39,40,41,42,43] revealed that 23% were categorized as low risk of bias and the remainder as moderate risk of bias; none of the included studies were considered to have a high risk of bias. The complete bias assessment table can be found in the Supplementary Materials (Table S1).

### 3.3. Patient’s Characteristics

We included a total of 2822 with a median age of 38.15 years (range, 47–63 years) [1,2,3,4,5,10,11,12,13,14,15,16,17,18,19,20,21,22,23,24,25,26,27,28,29,30,31,32,33,34,35,36,37,38,39,40,41,42,43]; the relevant characteristics of the included studies are reported in Table 1.

All patients had an LMC diagnosis; the most common underlying primary cancers in the cohort were lung cancer (*n* = 1337) and breast cancer (*n* = 990), followed by gastric cancer (*n* = 102), hematopoietic tumors (*n* = 84), and melanoma (*n* = 46) [1,2,3,4,5,10,11,12,13,14,15,16,17,18,19,20,21,22,23,24,25,26,27,28,29,30,31,32,33,34,35,36,37,38,39,40,41,42]. Less frequent tumor types included prostatic (*n* = 11), esophageal (*n* = 11), head and neck tumors (*n* = 8), central nervous system tumors (*n* = 6), ovarian (*n* = 5), renal (*n* = 4), pancreatic (*n* = 4), uterus (*n* = 3), and liver (*n* = 3), and there was only one reported case of peripheral nervous system (PNS), thyroidal, testicular, gall bladder, leukemia, germ cell tumor, and thymic tumors [1,2,3,4,5,10,11,12,13,14,15,16,17,18,19,20,21,22,23,24,25,26,27,28,29,30,31,32,33,34,35,36,37,38,39,40,41,42,43]. The type of radiotherapy used on the different kinds of primary cancers and the number of patients for each type are shown in Table 2. Tumor stage at initial diagnosis was frequently advanced (Stage III or IV), reflecting the aggressive nature of malignancies prone to leptomeningeal dissemination [12,18,19,24,31,32,35].

### 3.4. LMC Presentation and Treatment

The reported survival and neurological outcomes varied widely across studies and radiotherapy techniques and were therefore summarized descriptively without quantitative pooling. Across studies, radiotherapy was frequently associated with reported neurological stabilization or partial symptomatic improvement, particularly in patients treated for focal neurological deficits or cerebrospinal fluid flow obstruction. However, definitions of clinical response varied substantially, and formal, standardized symptom scales were rarely employed. Consequently, these outcomes were summarized descriptively and not pooled quantitatively. The mean time from primary cancer diagnosis to LMC diagnosis was 22.44 months (2–96.2 months) [1,2,3,4,5,10,11,12,13,14,15,16,17,18,19,20,21,22,23,24,25,26,27,28,29,30,31,32,33,34,35,36,37,38,39,40,41,42,43]. In some studies, patients were diagnosed with LMC and the primary tumor at the same time, so when enrolled in the study they already had both diagnoses [5,10,24,27,28,30,39]. The initial and final functional statuses were evaluated with scales such as Karnofsky, ECOG, GPA [1,2,3,4,5,10,11,12,13,14,15,16,17,18,19,20,21,22,23,24,25,26,27,28,29,30,31,32,33,34,35,36,37,38,39,40,41,42,43]. The baseline functional status was not uniformly reported and the performance status at LMC diagnosis was often described as moderate to poor, reflecting a high disease burden [2,3,11,14,19,21,25,28,30,32,35,39].

Across the included studies, various radiotherapy treatment strategies were employed, including WBRT in 1054 individuals, CSI in 148 individuals, and focal radiotherapy in 27 individuals [1,2,3,4,5,10,11,12,13,14,15,16,17,18,19,20,21,22,23,24,25,26,27,28,29,30,31,32,33,34,35,36,37,38,39,40,41,42,43]. In one study, the use of radiotherapy was described but the type was not specified (*n* = 33) [23]. All radiotherapy types were used both as monotherapy and in combination treatments [1,2,3,4,5,10,11,12,13,14,15,16,17,18,19,20,21,22,23,24,25,26,27,28,29,30,31,32,33,34,35,36,37,38,39,40,41,42,43]. Finally, the mean LMC treatment duration was 323.43 months (2–789 months) [1,4,10,11,13,14,15,16,17,18,21,22,25,29,30,31,34,36,39,41] with a mean dose of RT of 32.01 Gy (27.5–30 Gy) [2,3,5,10,11,13,14,17,19,25,26,29,30,31,33,34,36,38,40].

### 3.5. Overall Survival and Response to Treatment

In the studies, the OS was described both from the primary cancer diagnosis as from LMC diagnosis, we decided to focus on the OS from LMC diagnosis, which is described in 37 articles [1,2,3,4,5,10,11,12,13,14,15,16,17,19,20,21,22,23,24,25,26,27,28,29,30,31,32,33,34,35,36,37,38,40,41,42,43]. Those patients received different types of treatments that included radiotherapy alone, chemotherapy alone, or a combined treatment scheme [1,2,3,4,5,10,11,12,13,14,15,16,17,19,20,21,22,23,24,25,26,27,28,29,30,31,32,33,34,35,36,37,38,40,41,42,43]. The OS, regardless of the type of therapy used, from LMC diagnosis to death of any cause ranged from 4 to 62.85 weeks, with a mean OS of 18.17 weeks and a median of 14.08 weeks [1,2,3,4,5,10,11,12,13,14,15,16,17,19,20,21,22,23,24,25,26,27,28,29,30,31,32,33,34,35,36,37,38,40,41,42,43]. The OS in relation to the type of treatment was detailed in 32 articles, in which we could determine the difference between patients that received only radiotherapy, other types of treatment as systemic chemotherapy or intrathecal chemotherapy, and combined therapy [2,3,4,5,10,11,13,14,17,19,20,21,22,23,24,25,26,27,28,29,30,31,33,34,35,36,37,38,40,41,42,43]. Mean OS for radiotherapy alone was 21.48 weeks, for combined therapy was 20.28 weeks, and for non-radiotherapy treatments was 23.5 weeks [2,3,4,5,10,11,13,14,17,19,20,21,22,23,24,25,26,27,28,29,30,31,33,34,35,36,37,38,40,41,42,43]. However, the longest reported OS in all articles was 62.85 weeks with WBRT alone [29]. The reported ranges for the OS were from 6 to 62.85 weeks for WBRT, from 16 to 28 weeks for combined therapy, and from 8.4 to 46.8 weeks for non-radiotherapy treatments [2,3,4,5,10,11,13,14,17,19,20,21,22,23,24,25,26,27,28,29,30,31,33,34,35,36,37,38,40,41,42,43]. However, the observed differences in overall survival between treatment groups should be interpreted descriptively and not as evidence of comparative effectiveness, given the non-randomized nature of the data and substantial confounding by indication.

There were other contributing factors that reportedly affected the OS time such as the type of cancer, the availability of targeted therapy, and the functional state prior to treatment [2,3,4,5,10,11,13,14,17,19,20,21,22,23,24,25,26,27,28,29,30,31,33,34,35,36,37,38,40,41,42,43]. There were 10 studies that concluded that better treatment response resulted from combining radiotherapy with systemic or intrathecal chemotherapy [1,10,17,21,23,24,26,32,40,43]. On the other hand, nine studies did not show a statistically significant difference in the OS when combining treatments [5,13,14,15,25,28,31,33,38], and in most studies [12], it was shown that other factors such as the primary tumor, the patient’s age, or the ECOG performance status affected the OS time more than the type of therapy being used [2,5,10,11,17,19,20,22,29,35,36,41].

### 3.6. Radiotherapy Toxicity: Meta-Analysis of Proportions

Radiotherapy toxicity was described in 16 articles, with a total of 462 patients suffering from adverse effects [2,3,11,15,16,19,26,28,29,31,34,36,39,40,41,43]; however, only 12 articles had enough granular information to properly dichotomize toxicity for our proportions meta-analysis [2,3,15,19,26,28,29,31,34,36,39,43].

The most used scale to describe the toxicity effects was the CTCAE scale, used to qualify the type of toxicity in nine different studies [2,3,19,28,29,31,36,39,43]. According to that scale, most adverse events caused by radiotherapy were Grade 1, with fatigue being the most prevalent event [2,3,31,39]. A total of 115 patients presented fatigue during radiotherapy, seven with WBRT, 40 with CSI, and 68 with other types of non-specified radiotherapy [2,3,31,39]. Table 3 showcases the absolute frequency of adverse effects according to the type of radiotherapy, organized from the most to least common. Most patients that suffered from Grade 3 or 4 adverse events such as thrombocytopenia, leukopenia, anemia, and lymphopenia were receiving CSI [3,26,34,39]. However, neurological adverse effects, such as leukoencephalopathy and seizures, were only present in patients who received WBRT, even though the cases were scarce (seven in total) [15,29]. Cutaneous toxicity was also more common in patients treated with WBRT, with seven cases of skin erythema and 12 cases of radiation dermatitis [2,19,28,31,43]. The most common effects in WBRT patients were nausea, vomiting and headache [2,3,11,15,19,29,31,36,38,40,43]. The greatest number of patients for whom an adverse effect was described received CSI, with a total 220 cases, and most effects were hematological toxicities [2,3,26,34,39]. These results are relevant, as there were more patients that received WBRT (*n* = 1054) than CSI (*n* = 148) [2,3,11,15,16,19,26,28,29,31,34,36,39,40,41,43].

**Figure 2:**
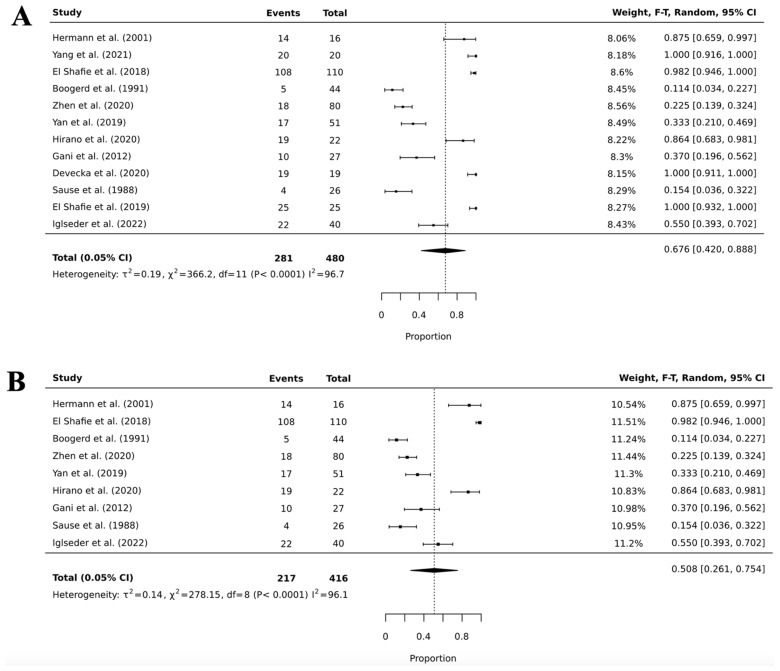
Forest plots of all radiotherapy pooled toxicity prevalence before (panel (**A**)) [2,3,15,19,26,28,29,31,34,36,39,43] and after (panel (**B**)) sensitivity analysis [3,15,19,26,28,29,31,36,43].

When looking at the pooled prevalence of toxicity obtained in our mixed-effects proportions meta-analysis (Figure 2), we found a 67.6% (95% CI, 42.0–88.8; I^2^ = 96.7; *p* < 0.0001) pooled toxicity prevalence for all RT modalities (i.e., WBRT, CSI, and unspecified); however, after conducting a sensitivity analysis that involved the removal of outliers, we obtained a pooled toxicity prevalence for all RT modalities of 50.8% (95% CI, 26.1–75.4; I^2^ = 96.1; *p* < 0.0001). It is important to note that both results had significant heterogeneity with very high I^2^ values.

**Figure 3:**
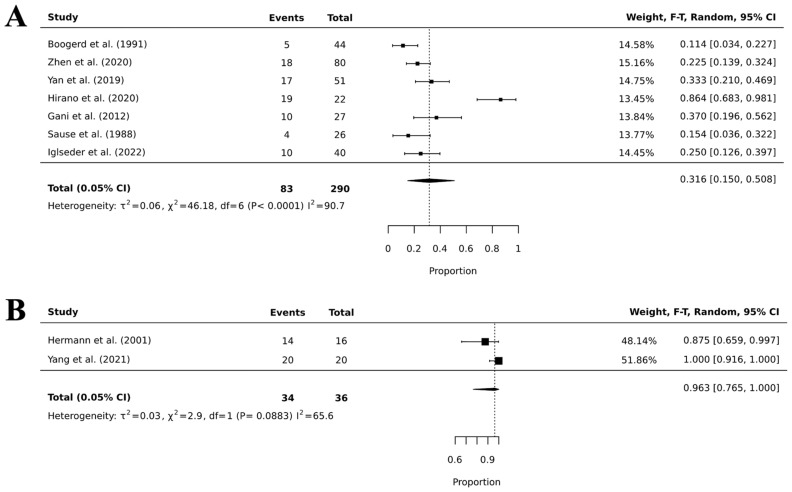
Forest plots of pooled toxicity prevalence for WBRT (panel (**A**)) [15,19,28,29,31,36,43] and CSI (panel (**B**)) [26,39].

We also calculated the pooled prevalence of WBRT and CSI (Figure 3); for WBRT and CSI, we obtained a pooled toxicity prevalence of 31.6% (95%CI, 15.0–50.8; I^2^ = 90.7; *p* < 0.0001) and of 96.3% (95%CI, 76.5–100.0; I^2^ = 65.6; *p* = 0.08). The latter had a very high *p* value and low statistical power (i.e., these results are non-significant) because only two articles had enough granular information on CSI toxicity for us to be able to perform our meta-analysis. The pooled toxicity estimates were associated with substantial statistical heterogeneity (I^2^ > 90%), reflecting marked variability in study populations, radiotherapy techniques, dose and fractionation, toxicity definitions, and reporting practices. Accordingly, these pooled proportions should not be interpreted as precise or definitive risk estimates; rather, they are presented as exploratory summaries intended to characterize the overall burden and dispersion of reported toxicity across the literature, providing contextual information for clinical decision-making and patient counseling rather than formal comparative inference.

## 4. Discussion

Our systematic review synthesizing outcomes of radiotherapy in LMC highlights the continued importance of RT as a palliative intervention, despite the limited impact on survival. Across 39 studies and over 2800 patients, we found that overall survival remains importantly short, of only a few months from LMC diagnosis, which is consistent with historical reports of median OS of approximately 10–15 weeks [3]. Notably, we observed no significant difference in OS between patients receiving RT alone and those receiving combined modality therapy (approximately 21.5 vs. 20.3 weeks, respectively). This aligns with prior observations that involved-field RT (e.g., WBRT) can provide symptomatic relief but generally does not prolong survival in LMC [6,7,8]. For example, a retrospective study in EGFR-mutant NSCLC LMC found that adding WBRT did not improve survival compared to systemic therapy alone [38]. These findings reinforce that RT’s value in LMC lies primarily in palliation rather than extending life, and survival outcomes appear to be driven more by tumor biology and patient factors than by the use of radiotherapy [6,8].

Our analysis offers insight into the relative benefits and drawbacks of different RT modalities in LMC. Whole-brain RT was by far the most commonly employed approach, reflecting its utility in managing diffuse leptomeningeal disease and multifocal symptoms [1,2,3,4,5,10,11,12,13,14,15,16,17,18,19,20,21,22,23,24,25,26,27,28,29,30,31,32,33,34,35,36,37,38,39,40,41,42,43]. Craniospinal irradiation, in contrast, was used in only a small subset (under 5% of patients), likely due to concerns about toxicity and the need for careful patient selection [1,2,3,4,5,10,11,12,13,14,15,16,17,18,19,20,21,22,23,24,25,26,27,28,29,30,31,32,33,34,35,36,37,38,39,40,41,42,43]. Focal radiotherapy to symptomatic sites was even less common, reserved for isolated bulky deposits or specific pain control, and thus its impact on overall disease control is difficult to generalize [1,2,3,4,5,10,11,12,13,14,15,16,17,18,19,20,21,22,23,24,25,26,27,28,29,30,31,32,33,34,35,36,37,38,39,40,41,42,43]. Importantly, we found that radiotherapy-associated adverse events, while not rare, were usually mild to moderate in severity. Approximately 15% of patients in the included studies experienced RT-related toxicity, most frequently fatigue (the single most reported symptom) and gastrointestinal disturbance (nausea/vomiting) [1,2,3,4,5,10,11,12,13,14,15,16,17,18,19,20,21,22,23,24,25,26,27,28,29,30,31,32,33,34,35,36,37,38,39,40,41,42,43]. The prevalence and profile of toxicity differed by RT modality; conventional CSI was associated with substantially higher hematological toxicity, notably myelosuppression [7]. In our review, hematological adverse events were predominantly seen in CSI-treated patients, reflecting irradiation of extensive bone marrow reserves in the spine. In fact, prior institutional series report that up to one-third of adults receiving CSI for LMC develop Grade 3 or worse cytopenias [2]. In our meta-analysis, CSI pooled toxicity could not be calculated due to a lack of studies reporting enough granular data to properly conduct a meta-analysis; this also highlights an important downfall of the reported cancer therapy research: the lack of reported patient-specific granular information that allows researchers to disaggregate relevant outcomes, such as toxicity, in order to perform informative and impactful meta-analytical statistics. On the other hand, WBRT tended to produce more neurological side effects (such as cognitive impairment or exacerbation of intracranial pressure symptoms), but had a more limited impact on systemic toxicity [7]. We calculated a pooled toxicity prevalence for WBRT of 31.6% (95%CI, 15.0–50.8; I^2^ = 90.7; *p* < 0.0001), although with very high heterogeneity. These modality-specific differences underscore the trade-offs in choosing a radiotherapy strategy; CSI offers the theoretical benefit of treating the entire neuraxis (potentially addressing diffuse disease), yet at the cost of greater toxicity, whereas focal or whole-brain treatments are better tolerated but leave a risk of out-of-field progression. Overall, our findings suggest that RT for LMC is generally safe and manageable in a palliative context, with careful attention needed to modality-related side effect profiles. Notably, serious complications were infrequent in the literature, especially when modern techniques (e.g., IMRT or helical tomotherapy) were employed to minimize normal tissue dosing [2].

Our study contributes to the understanding of RT’s role in LMC by consolidating outcomes across a wide range of cancer types, treatment approaches, and clinical settings. Despite the lack of a clear survival advantage, radiotherapy remains useful for LMC management due to its ability to stabilize or improve neurological symptoms and quality of life [6,7]. Clinical practice guidelines underscore that in patients with reasonable performance status, RT should be considered to palliate symptoms such as cranial neuropathies, pain, or CSF flow obstruction, and to reduce bulky leptomeningeal tumors [6]. Our results support this paradigm; we observed that many patients derived symptomatic benefit from RT (as evidenced by the documented neurological improvement or stabilization in several series), even if tumor progression eventually continued [1,2,3,4,5,10,11,12,13,14,15,16,17,18,19,20,21,22,23,24,25,26,27,28,29,30,31,32,33,34,35,36,37,38,39,40,41,42,43]. In particular, the approximately 40–70% rate of symptom stabilization/improvement reported after CSI in select cohorts [2] speaks to the palliative efficacy of RT when applied to well-chosen patients. Thus, the primary contribution of RT in LMC is in improving neurological outcomes and providing disease control within the CNS compartment, rather than curing the disease. We also shed light on how patient-specific factors mediate the effectiveness of RT; consistent with prior studies, better functional status (e.g., Karnofsky ≥ 60–70) and controlled systemic disease were associated with longer survival and better responses [1,2,3,4,5,6,8,10,11,12,13,14,15,16,17,18,19,20,21,22,23,24,25,26,27,28,29,30,31,32,33,34,35,36,37,38,39,40,41,42,43]. In other words, patients who are less debilitated at LMC presentation tend to benefit most from any active therapy, including radiotherapy. This reinforces the importance of careful patient selection, considering factors such as performance status, extent of systemic cancer, and tumor molecular subtype when deciding on aggressive treatments like CSI or combined modality therapy. Indeed, it appears that prognosis in LMC is more strongly influenced by patient and disease factors (primary tumor type, tumor burden, ECOG/KPS, age) than by the choice of any specific treatment regimen [8]. This observation may explain why radiotherapy trials to date have struggled to demonstrate a survival benefit given that the underlying biology often dictates outcomes regardless of intervention. However, it is very important to highlight that the high heterogeneity observed in the toxicity meta-analysis warrants careful interpretation. In the context of rare conditions such as leptomeningeal metastases, where prospective data are scarce and reporting practices are inconsistent, pooled proportion estimates primarily serve an exploratory function. Their clinical interpretability lies in illustrating the magnitude and variability of treatment-related toxicity rather than in defining precise incidence rates. Consequently, these findings should be viewed as hypothesis-generating and descriptive, highlighting uncertainty and informing risk–benefit discussions rather than guiding definitive treatment selection. Furthermore, the concept of radiotherapy effectiveness in leptomeningeal disease must be interpreted cautiously. While several studies reported neurological stabilization or symptomatic improvement following radiotherapy, these outcomes were inconsistently defined and infrequently quantified using standardized measures. As such, the present review does not support definitive quantitative conclusions regarding symptomatic benefit. Instead, it highlights that radiotherapy may provide clinically meaningful symptom control in selected patients, based on descriptive evidence, while underscoring the need for future studies incorporating validated neurological and quality-of-life endpoints.

### 4.1. Limitations

Several limitations in the existing literature and in our analysis must be acknowledged. First, there is significant heterogeneity among the included studies. The reports span different primary malignancies (with breast and lung dominating, but also melanoma, GI, etc.), various radiotherapy techniques/doses, and a range of concomitant therapies (from intrathecal chemotherapy to newer targeted agents). This heterogeneity made direct comparisons challenging and precluded any meta-analysis of efficacy beyond broad metrics and proportions meta-analysis for toxicities. Furthermore, endpoints were not standardized; while most studies reported OS, some measured it from LMC diagnosis and others from the start of RT and the definitions of neurological response and functional improvement varied. Only a subset of studies systematically documented toxicities or quality-of-life outcomes. The lack of uniform reporting, for instance, inconsistent documentation of performance status or neurological exam findings, limited our ability to draw nuanced conclusions about prognostic factors across studies. Additionally, many of the data are derived from retrospective single-institution series with inherent biases. Patients selected for intensive therapies like CSI were often those with longer expected survival or better condition, which could skew comparisons. Conversely, patients with poor prognosis may have been underrepresented in interventional cohorts. Furthermore, baseline patient characteristics, particularly performance status, represent a major source of confounding in survival interpretation. Performance status is a well-established prognostic factor in leptomeningeal disease; however, its reporting was inconsistent across the included studies, with heterogeneous scales and frequent omission. This limitation substantially restricts the comparability of the reported survival outcomes and reinforces that observed differences should not be interpreted as treatment-related effects. Future studies would benefit from standardized reporting of the baseline performance status and neurological function to enable more robust comparative analyses. The evidence base also contains only a few small prospective studies and no recent randomized trials specifically evaluating RT in LMC, meaning that the overall level of evidence is low. Notably, one of the only randomized efforts in this field, a 1988 SWOG study of WBRT plus intrathecal methotrexate, failed to show a clear survival benefit, illustrating the difficulty of improving outcomes in this disease [36]. Another important limitation is the limited sample size for certain RT modalities. For example, our review identified only 148 patients who received CSI and merely 27 who received focal RT alone, numbers too small to allow robust statistical analysis or subgroup meta-analysis, as discussed previously. As a result, conclusions regarding these modalities must be tentative. Furthermore, the included studies varied in their definition of leptomeningeal disease, with some requiring cytological confirmation and others relying on MRI-based diagnostic criteria. This variability may have contributed to between-study heterogeneity in the reported outcomes and limits direct comparability across cohorts. As such, findings should be interpreted with caution, and future studies would benefit from more standardized diagnostic frameworks integrating cytological, radiographic, and clinical criteria. Encouragingly, some recent studies have begun to propose prognostic scoring systems to guide RT use (such as a risk–benefit scores for CSI in the palliative setting [34]), but these need validation. In general, the current literature provides a broad, qualitative understanding of how RT can be used in LMC, but it lacks the precision and consistency that high-quality, controlled studies would offer; this is a critical gap in knowledge that our systematic review brings to attention and that we should strive to fulfill in future endeavors.

### 4.2. Implications and Future Directions

Going forward, our findings underscore that radiotherapy should be viewed as one component of a multimodal approach to LMC, tailored to the individual patient’s condition. While RT alone is unlikely to alter the trajectory of LMC, it remains a key palliative tool that can improve neurological function or temporarily control disease within the CNS [7], thereby potentially enabling patients to better tolerate or bridge to systemic treatments. The choice of RT modality and extent (WBRT, partial brain/spine, or CSI) should be individualized based on the patient’s overall prognosis, burden of leptomeningeal disease, and ability to tolerate treatment. For instance, CSI may be considered in a “good-risk” patient (e.g., higher KPS, minimal systemic disease) with extensive neuraxial involvement, especially if delivered with advanced techniques to mitigate toxicity [7]. In contrast, a frail patient may derive more benefit from focused RT to dominant symptomatic sites, avoiding undue toxicity. Our review highlights an urgent need for prospective research to clarify the optimal role of RT in the modern era of LMC management [6,8]. Future studies should strive to stratify patients by tumor subtype and risk factors, and ideally randomize or compare RT approaches within defined subgroups. Given the advent of more effective systemic therapies (e.g., targeted tyrosine kinase inhibitors and intrathecal antibodies) that can penetrate the CNS, it will be important to evaluate how radiotherapy can be combined with these treatments to maximize overall disease control.

Certainly, recent studies in leptomeningeal carcinomatosis indicate an increasing role for targeted systemic and intrathecal therapies as alternatives or complements to conventional chemotherapy and radiotherapy, particularly in molecularly defined subgroups [44,45,46,47,48,49]. Third-generation EGFR tyrosine kinase inhibitors, notably osimertinib and aumolertinib, demonstrate superior central nervous system penetration and clinically meaningful improvements in overall survival, progression-free survival, and disease control compared with earlier-generation agents [47,49]. The reported median overall survival ranges from 15.6 to 17.7 months, with intracranial or overall progression-free survival approaching 9.7–11.3 months, and overall response rates exceeding 50%, alongside leptomeningeal disease control rates above 80% [47,49]. Importantly, treatment benefit appears to be strongly modulated by the baseline functional status, with lower ECOG performance scores consistently associated with improved survival outcomes, reinforcing the prognostic importance of patient selection in LMC management [47,49]. Similarly, HER2-targeted therapies have shown promising activity in HER2-positive breast cancer with leptomeningeal involvement. Trastuzumab deruxtecan has been associated with progression-free survival of up to 14.6–16.1 months and intracranial response rates exceeding 60%, with a subset of patients achieving overall survival approaching two years [45,46]. Intrathecal trastuzumab has also demonstrated clinical benefit, including neurological symptom improvement, albeit with more modest survival outcomes [44]. Across studies, toxicity profiles were generally acceptable, though clinically relevant adverse events such as interstitial lung disease with trastuzumab deruxtecan and transient neurological symptoms with intrathecal administration were reported [44,45]. While the present work focuses on radiotherapy in LMC, these data highlight that targeted therapies offer meaningful disease control in selected populations and provide an important comparative context for evaluating the palliative and therapeutic role of radiotherapy in leptomeningeal carcinomatosis in the future [44,45,46,47,48,49].

## 5. Conclusions

Leptomeningeal carcinomatosis remains a devastating complication of advanced malignancies, most frequently arising from lung and breast cancers, and is characterized by profound neurological morbidity and limited survival. In this systematic review including over 2800 patients, overall survival following LMC diagnosis remained poor across all treatment strategies, with no consistent survival advantage attributable to the radiotherapy modality or treatment combination, reinforcing that prognosis is driven predominantly by patient performance status, primary tumor biology, and systemic disease burden rather than by radiotherapy itself. Nevertheless, radiotherapy continues to play a palliative role, particularly for symptom control and neurological stabilization. However, it is important to note that radiotherapy may offer symptomatic relief or neurological stabilization in selected patients, based on the descriptive evidence, although quantitative data on clinical improvement remain limited. Our meta-analysis demonstrated a pooled prevalence of radiotherapy-related toxicity, estimated at 67.6% before sensitivity analysis and 50.8% after outlier removal, with marked heterogeneity across studies. Modality-specific analyses showed a lower pooled toxicity prevalence for whole-brain radiotherapy (31.6%) compared with craniospinal irradiation (96.3%; non-significant), the latter being predominantly associated with hematological adverse events. These findings underscore the need for careful patient selection and individualized treatment planning, balancing the expected palliative benefit against the toxicity risk. Overall, radiotherapy remains an important tool in LMC management within a palliative framework; however, our review highlights the heterogeneity of the existing evidence and underscores that the current data primarily support descriptive conclusions regarding efficacy, while pooled estimates can be generated for toxicity outcomes. These findings may inform clinical decision-making and future prospective studies, but do not support definitive comparative effectiveness conclusions between radiotherapy modalities.

## Figures and Tables

**Table 1:** Relevant characteristics of the 39 included studies.

N° (Ref)	Country	Type of Study	Number of Patients	Mean Age (Years)	Type of Cancer	Median Time from Cancer Diagnosis to LMC (Months)	LMC Treatment
1 [37]	USA	Retrospective cohort study	9	57.1	Colorrectal	25.3	Systemic + RT
2 [24]	South Korea	Retrospective cohort study	54	48.5	Gastric	6.3	Systemic + RT
3 [39]	USA	Clinical trial	24	52	Various	Enrolled with LMC	Systemic + RT
4 [23]	USA	Retrospective cohort study	33	NR	Various	NR	Systemic + RT
5 [28]	China	Retrospective cohort study	136	55	Various	Enrolled with LMC	Systemic + RT
6 [36]	USA	Prospective cohort study	26	54	Various	NR	Systemic + RT
7 [38]	China	Retrospective cohort study	51	56	Lung	17.4	Systemic + RT
8 [19]	China	Retrospective cohort study	80	53.5	Lung	17.6	Systemic + RT
9 [20]	Spain	Retrospective cohort study	38	NR	Breast	59.55	Systemic + RT
10 [2]	Germany	Retrospective cohort study	25	NR	Various	25	Systemic + RT
11 [3]	Germany	Retrospective cohort study	110	NR	Various	60.4	RT
12 [4]	Turkey	Retrospective cohort study	16	53	Various	6	Systemic + RT
13 [18]	China	Retrospective cohort study	38	45	Various	NR	Systemic + RT
14 [21]	Korea	Retrospective cohort study	58	51	Various	18.4	Systemic + RT
15 [31]	Germany	Retrospective cohort study	27	57	Various	35	Systemic + RT
16 [30]	USA	Retrospective cohort study	21	51	Gastrointestinal	NR	Systemic + RT
17 [29]	Japan	Retrospective cohort study	22	60	Various	NR	Systemic + RT
18 [27]	Scotland	Retrospective cohort study	36	51	Various	NR	Systemic + RT
19 [42]	Korea	Retrospective cohort study	519	56	Various	18	Systemic + RT
20 [1]	USA	Retrospective cohort study	103	48	Breast	NR	Systemic + RT
21 [40]	South Korea	Retrospective cohort study	71	60	Various	4	Systemic + RT
22 [12]	Korea	Retrospective cohort study	9	53	Gastric	10	Systemic + RT
23 [16]	Greece	Retrospective cohort study	155	54	Breast	6	Systemic + RT
24 [35]	Italy	Retrospective cohort study	153	50	Breast	37.55	Systemic + RT
25 [26]	Germany	Retrospective cohort study	16	46	Various	5	Systemic + RT
26 [43]	Austria	Retrospective cohort study	40	59	Various	NR	Systemic + RT
27 [5]	Germany	Retrospective cohort study	135	54.4	Various	12	Systemic + RT
28 [32]	USA	Retrospective cohort study	153	55.7	Various	24	Systemic + RT
29 [15]	Netherlands	Prospective cohort study	58	NR	Breast	NR	Systemic + RT
30 [34]	Germany	Retrospective cohort study	19	57.8	Various	NR	RT
31 [22]	Brazil	Retrospective cohort study	60	46	Breast	17.9	Systemic + RT
32 [41]	Taiwan	Retrospective cohort study	34	60	Lung	Enrolled with LMC	Systemic + RT
33 [17]	USA	Retrospective cohort study	124	52	Various	8.7	Systemic + RT
34 [33]	USA	Retrospective cohort study	12	NR	Lung	13	Systemic + RT
35 [11]	USA	Retrospective cohort study	51	53	Lung	13.2	RT
36 [14]	USA	Retrospective cohort study	59	59	Lung	15	Systemic + RT
37 [25]	China	Retrospective cohort study	184	57	Lung	13.3	Systemic + RT
38 [13]	Japan	Retrospective cohort study	31	58	Breast	NR	Systemic + RT
39 [10]	USA	Prospective cohort study	32	57	Lung	7.5	Systemic + RT

LMC = Leptomeningeal Carcinomatosis. RT = Radiotherapy, NR = Not reported.

**Table 2:** Number of patients for each type of primary tumor and RT used [1,2,3,4,5,10,11,12,13,14,15,16,17,18,19,20,21,22,23,24,25,26,27,28,29,30,31,32,33,34,35,36,37,38,39,40,41,42,43].

Primary Tumor	N° Patients	Type of RT
Lung	1337	WBRT, CSI
Breast	990	WBRT, CSI
Gastric	102	WBRT, CSI
Non-specified hematopoyetic	84	WBRT, CSI
Non-specified GI	69	WBRT, CSI
Melanoma	46	WBRT, CSI
Colorectal	20	WBRT
Prostatic	11	WBRT, CSI
Esophageal	11	WBRT, CSI
Head and neck	8	WBRT, CSI
CNS	6	CSI
Lymphoma	5	WBRT, CSI
Ovarian	5	WBRT, CSI
Renal	4	WBRT, CSI
Pancreas	4	WBRT, CSI
Uterus	3	WBRT, CSI
Liver	3	WBRT, CSI
Parotideal	2	CSI
Sarcoma	2	CSI
Bladder	2	WBRT, CSI
PNS	1	CSI
Thyroideal	1	WBRT, CSI
Gall bladder	1	WBRT, CSI
Leukemia	1	CSI
Germ cell	1	WBRT, CSI
Thymus	1	WBRT, CSI
Testicular	1	WBRT, CSI
Non-specified	99	WBRT, CSI
Total	2822	

CNS: central nervous system; CSI: craniospinal irradiation; GI: gastrointestinal; PNS: peripheral nervous system; WBRT: whole-brain radiotherapy.

**Table 3:** Absolute frequencies of toxicities in patients receiving different types of RT [2,3,11,15,16,19,26,28,29,31,34,36,39,40,41,43].

Type of Toxicity	WBRT	CSI	Non-Specified RT	Total
Fatigue	7	40	68	115
Nausea/vomiting	23	17	32	72
Myelosuppresion		19	19	38
Headache	23	7		30
Thrombocitopenia		28		28
Leukopenia		24		24
Mucositis/dysphagia		21		21
Skin erythema	7	7	7	21
Lymphopenia		18		18
Radiation dermatitis	12	6		18
Alopecia	15			15
Anemia		11		11
Non-specified hematological toxicity	6			6
Anorexia	4	1		5
Neutropenia		5		5
Leukoencephalopathy	5			5
Dysgeusia		3		3
Tinnitus	3			3
Xerostomia		3		3
Somnolence	3			3
Dry skin		2		2
Seizures	2			2
Candida infection		2		2
Visual field restriction	2			2
Pain		1		1
Dyspepsia		1		1
Back pain		1		1
Gait disturbance		1		1
Memory impairment		1		1
Sensory impairment		1		1
Hearing impairment	1			1
Dizziness	1			1
Malaise	1			1
External otits	1			1
Total	116	220	126	462

WBRT = Whole Brain Radiation Therapy, CSI = Craniospinal Irradiation, RT = Radiotherapy.

## Data Availability

All data are available in the manuscript.

## Supplementary Materials

The following supporting information can be downloaded at https://www.mdpi.com/article/10.3390/cancers18040547/s1, Table S1: The complete bias assessment table [1,2,3,4,5,10,11,12,13,14,15,16,17,18,19,20,21,22,23,24,25,26,27,28,29,30,31,32,33,34,35,36,37,38,39,40,41,42,43].
